# Supplementary material for: Mechanisms governing the pioneering and redistribution capabilities of the non-classical pioneer PU.1
Source: Nat Commun. 2020 Jan 21;11:402. doi: 10.1038/s41467-019-13960-2 (PMC6972792; doi:10.1038/s41467-019-13960-2)
Supplement: Supplementary file 7 — Source data [file 41467_2019_13960_MOESM7_ESM.zip › Source_Data/FigureS2/FigureS2B_MotifScanOutput/KmeansCluster1/homerResults/motif26.similar.html]

motif26

## Information for motif26

A
G
C
T
A
G
C
T
G
A
C
T
G
T
C
A
A
C
G
T
T
G
A
C
C
G
T
A
C
T
A
G
C
G
T
A
A
C
G
T
T
G
A
C
C
G
A
T
C
G
T
A
  
Reverse Opposite:  

A
C
G
T
C
G
T
A
A
C
T
G
G
T
C
A
G
C
A
T
G
A
T
C
C
G
A
T
A
C
T
G
C
G
T
A
A
C
G
T
C
T
G
A
T
C
G
A
T
C
G
A
  

|  |  |
| --- | --- |
| p-value: | 1e-31 |
| log p-value: | -7.147e+01 |
| Information Content per bp: | 1.775 |
| Number of Target Sequences with motif | 55.0 |
| Percentage of Target Sequences with motif | 1.08% |
| Number of Background Sequences with motif | 29.6 |
| Percentage of Background Sequences with motif | 0.07% |
| Average Position of motif in Targets | 84.9 +/- 75.6bp |
| Average Position of motif in Background | 81.8 +/- 50.9bp |
| Strand Bias (log2 ratio + to - strand density) | 0.5 |
| Multiplicity (# of sites on avg that occur together) | 1.00 |
| Motif File: | file (matrix) reverse opposite |

### Similar de novo motifs found

|  |  |  |  |  |  |  |  |
| --- | --- | --- | --- | --- | --- | --- | --- |
| Rank | Match Score | Redundant Motif | P-value | log P-value | % of Targets | % of Background | Motif file |
| 1 | 0.938 | A C G T C G A T C T G A A C G T A T G C C G T A C T A G C G T A C G A T G A T C A C G T C G T A | 1e-24 | -56.857735 | 0.84% | 0.06% | motif file (matrix) |
| 2 | 0.895 | G A C T T G C A C G A T T A G C C G T A C T A G C G A T A C G T A G T C A C G T C G T A | 1e-23 | -54.089573 | 1.10% | 0.14% | motif file (matrix) |
